# Isotope ratio-based quantification of carbon assimilation highlights the role of plastidial isoprenoid precursor availability in photosynthesis

**DOI:** 10.1186/s13007-021-00731-8

**Published:** 2021-03-30

**Authors:** Matthew E. Bergman, Diego González-Cabanelas, Louwrance P. Wright, Berkley J. Walker, Michael A. Phillips

**Affiliations:** 1 Department of Cell and Systems Biology, University of Toronto, Toronto, ON M5S 3G5 Canada; 2 Department of Biochemistry, Max Planck Institute for Chemical Ecology, 07745 Jena, Germany; 3 Zeiselhof Research Farm, Menlo Park, P.O. Box 35984, Pretoria, 0102 South Africa; 4 Department of Energy, Plant Research Laboratory, Michigan State University, East Lansing, MI 48824 USA; 5 Department of Plant Biology, Michigan State University, East Lansing, MI 48824 USA; 6 Department of Biology, University of Toronto-Mississauga, Mississauga, ON L5L 1C6 Canada

**Keywords:** Photosynthetic carbon assimilation, *Arabidopsis thaliana*, Stable isotope labeling, Fosmidomycin, Clomazone, Isotope ratio mass spectrometry, 2*C*-methyl-d-erythritol 4-phosphate pathway, Isoprenoid metabolism

## Abstract

**Background:**

We report a method to estimate carbon assimilation based on isotope ratio-mass spectrometry (IRMS) of ^13^CO_2_ labeled plant tissue. Photosynthetic carbon assimilation is the principal experimental observable which integrates important aspects of primary plant metabolism. It is traditionally measured through gas exchange. Despite its centrality in plant research, gas exchange performs poorly with rosette growth habits typical of *Arabidopsis thaliana*, mutant lines with limited biomass, and accounts poorly for leaf shading.

**Results:**

IRMS-based carbon assimilation values from plants labeled at different light intensities were compared to those obtained by gas exchange, and the two methods yielded similar values. Using this method, we observed a strong correlation between ^13^C content and labeling time (R^2^ = 0.999) for 158 wild-type plants labeled for 6 to 42 min. Plants cultivated under different light regimes showed a linear response with respect to carbon assimilation, varying from 7.38 nmol ^13^C mg^−1^ leaf tissue min^−1^ at 80 PAR to 19.27 nmol ^13^C mg^−1^ leaf tissue min^−1^ at 500 PAR. We applied this method to examine the link between inhibition of the 2*C*-methyl-d-erythritol-4-phosphate (MEP) pathway and suppression of photosynthesis. A significant decrease in carbon assimilation was observed when metabolic activity in the MEP pathway was compromised by mutation or herbicides targeting the MEP pathway. Mutants affected in MEP pathway genes 1-DEOXY-d-XYLULOSE 5-PHOSPHATE SYNTHASE (DXS) or 1-HYDROXY-2-METHYL-2-(E)-BUTENYL 4-DIPHOSPHATE SYNTHASE (HDS) showed assimilation rates 36% and 61% lower than wild type. Similarly, wild type plants treated with the MEP pathway inhibitors clomazone or fosmidomycin showed reductions of 52% and 43%, respectively, while inhibition of the analogous mevalonic acid pathway, which supplies the same isoprenoid intermediates in the cytosol, did not, suggesting inhibition of photosynthesis was specific to disruption of the MEP pathway.

**Conclusions:**

This method provides an alternative to gas exchange that offers several advantages: resilience to differences in leaf overlap, measurements based on tissue mass rather than leaf surface area, and compatibility with mutant *Arabidopsis* lines which are not amenable to gas exchange measurements due to low biomass and limited leaf surface area. It is suitable for screening large numbers of replicates simultaneously as well as post-hoc analysis of previously labeled plant tissue and is complementary to downstream detection of isotopic label in targeted metabolite pools.

**Supplementary Information:**

The online version contains supplementary material available at 10.1186/s13007-021-00731-8.

## Introduction

Photosynthetic carbon assimilation integrates many aspects of plant metabolism and environmental response into a single response variable. Carbon assimilation is typically determined through real-time measurements of gas exchange, which provide information on stomatal conductance, transpiration, intercellular CO_2_ concentration (Ci), and net carbon assimilation (A) via water vapor and CO_2_ gas analyzers [51]. These sensors are often combined with additional detector systems, such as online mass spectrometers, to provide information on the relative contributions of photorespiratory and non-photorespiratory sources of CO_2_ loss, which in turn inform efforts at improving crop productivity [15, 39, 52, 53]. Gas exchange measurements are the primary input into biochemical models of photosynthesis, which have yielded significant insights into the coupling of photosynthetic electron flow to chemical energy production through the carbon reduction cycle [4, 26, 87]. The accurate estimation of carbon assimilation is therefore central to understanding the photosynthetic response of plants to changes in CO_2_ concentration (i.e. *A/C*_*i*_ response curves) [75], water deficit [48], and salinity [27]. Accurate measurement of carbon assimilation is also a prerequisite for investigating different metabolic modes of the Calvin-Benson cycle, such as Rubisco-limited photosynthesis versus RuBP-regeneration-limited photosynthesis [75].

Measurements of carbon assimilation have been instrumental in resolving key aspects of central metabolism such as photorespiration [78–81]. Such measurements have also helped resolve the impact of biotic and abiotic stress on photosynthesis including heat [36, 71, 74], cold [2, 5, 10, 65], drought [48], and light stress [21]. Measurements of assimilation can also help reveal coordinated signaling transduction networks. Herbivory stress, for instance, generally inhibits photosynthesis rapidly and substantially, even in excess of what is predicted through loss of photosynthetically active leaf surface area [82, 86] (reviewed in [58]). The metabolic down regulation of photosynthesis in response to herbivory is thought to be accompanied by a shift to defensive metabolism, a process mediated by jasmonate and phytochrome B signaling [35]. Indeed, these growth-defense trade-offs can be uncoupled by relieving transcriptional suppression imposed by this regulatory pathway [17]. These findings highlight the importance of leaf carbon assimilation as a tool to understand fundamental questions of plant metabolism.

Despite the broad utility of gas exchange in measuring net carbon assimilation, limitations include low throughput and the difficulty of measuring plants with small leaves. Carbon assimilation is usually measured in tandem with water vapor fluxes in commercially available gas exchange systems that measure gas concentrations using infra-red gas analyzers (IRGAs) [14, 88]. These systems are well suited to measuring carbon assimilation on single leaves but require individual leaves (or plants) to be enclosed within a measurement cuvette or leaf clamp for extended periods of time to gather accurate measurements. Arrayed systems constructed to measure net carbon assimilation in multiple plants have been reported but are limited in throughput to the number of chambers and gas switching channels [30]. Their increased complexity also hampers more widespread adoption among researchers. While higher-throughput tools probing the light energy use efficiency of photosynthesis using chlorophyll fluorescence are available, direct measurements of gas exchange still offer the most direct quantification of carbon assimilation [6, 22, 72]. The limitations noted above restrict the high throughput use of assimilation measurements on a large number of plants with small leaf areas, which would be required to screen for or characterize mutants of model plants like *Arabidopsis thaliana* with impaired growth phenotypes. Such phenotypes are expected to occur when investigating mutations that disrupt metabolic networks that interact with photosynthesis.

**Fig. 1:**
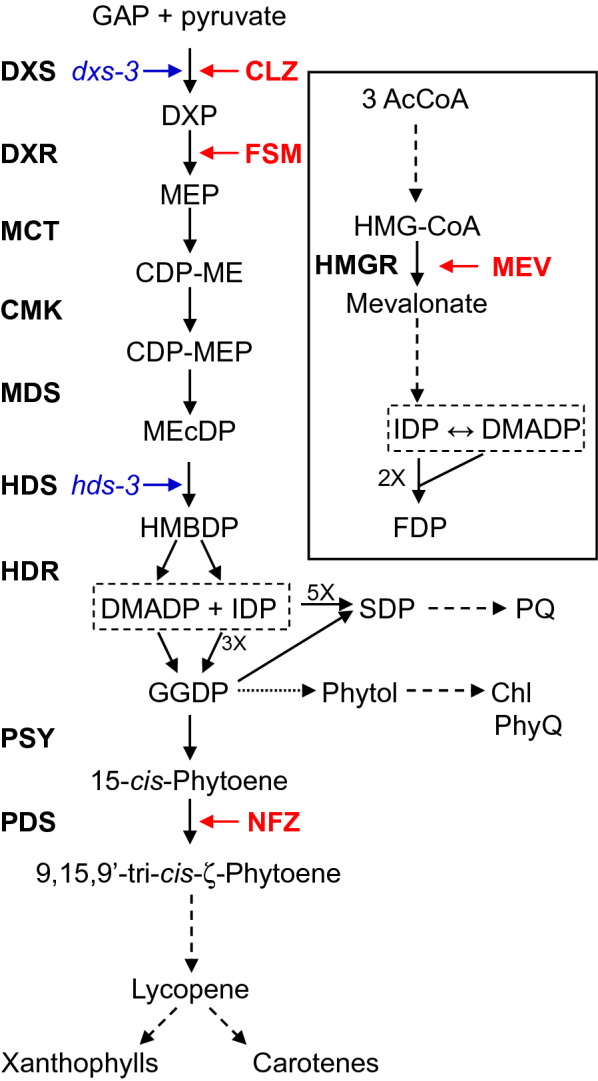
Involvement of the 2*C*-methyl-d-erythritol-4-phosphate (MEP) pathway in the biosynthesis of photosynthetic co-factors. The MEP pathway produces isopentenyl and dimethylallyl diphosphate (IDP and DMADP, boxed) in the plastid, which supplies PQ (plastoquinone), Chl (chlorophyll a and b), and PhyQ (phylloquinone) biosynthesis. Mutants (blue italics) and herbicides (red, bold) used in this study are shown next to the affected steps. Enzymes are shown in bold on the left. The mevalonate pathway, which yields IDP and DMADP in the cytosol, is enclosed in the solid box. The MEP pathway is directly linked to the formation of photosynthetic machinery by providing IDP and DMADP which are condensed into geranylgeranyl diphosphate (GGDP) by the prenyl transferase GGDP synthase (GGDS). GGDP undergoes three subsequent reductions to form phytyl diphosphate through dihydrogeranylgeranyl diphosphate and tetrahydrogeranylgeranyl diphosphate. Phytyl diphosphate provides the phytyl tail for phylloquinone and chlorophyll. The MEP pathway further supports photosynthesis through 8 total step-wise condensations of IDP with DMADP to form SDP, which becomes the hydrocarbon tail for plastoquinone. Herbicide abbreviations are as follows: CLZ, clomazone; FSM, fosmidomycin; NFZ, norflurazon; MEV, mevinolin. Biosynthetic intermediates are as follows: GAP, d-glyceraldehyde-3-phosphate; DXP, 1-deoxy-d-xylulose 5-phosphate; MEP, 2*C*-methyl-d-erythritol 4-phosphate; CDP-ME, 4-(cytidine 5′-diphospho)-2-*C*-methyl-d-erythritol; CDP-MEP, 4-(cytidine 5′-diphospho)-2-*C*-methyl-d-erythritol-2-phosphate; MEcDP, 2*C*-methyl-d-erythritol-2,4-cyclodiphosphate; HMBDP, 1-hydroxy-2-methyl-2-(*E*)-butenyl-4-diphosphate; SDP, solanesyl diphosphate; GGDP, geranylgeranyl diphosphate; AcCoA, acetyl-CoA; HMG-CoA, hydroxymethylglutaryl-CoA; FDP, farnesyl diphosphate. Enzymes abbreviations are as follows: DXS, 1-deoxy-d-xylulose 5-phosphate synthase; DXR, 1-deoxy-d-xylulose 5-phosphate reductoisomerase; MCT, 2C-methyl-d-erythritol 4-phosphate cytidyltransferase; CMK, 4-(cytidine 5′-diphospho)-2*C*-methyl-d-erythritol kinase; MDS, 2*C*-methyl-d-erythritol-2,4- cyclodiphosphate synthase; HDS, 4-hydroxy-3-methylbut-2-enyl diphosphate synthase; HDR, 4-hydroxy-3-methylbut-2-enyl diphosphate reductase; PSY, phytoene synthase; PDS, 15-*cis*-phytoene desaturase. Dotted arrows represent multiple steps

One metabolic domain closely linked to photosynthesis is that of chloroplastidic terpenoid (or isoprenoid) metabolism. All known terpenoids are synthesized from the universal precursors isopentenyl and dimethylallyl diphosphate (IDP and DMADP). IDP and DMADP are produced by two independent, compartmentally separated pathways in plants cells: the cytosolic mevalonic acid (MVA) pathway and the plastid localized 2*C*-methyl-d-erythritol-4-phosphate (MEP) pathway [63]. Efforts to dissect the regulatory mechanisms controlling these two pathways have relied heavily on specific inhibitors targeting enzymes of each pathway. Mevastatin (MEV) targets the rate determining step of the MVA pathway, 3-hydroxy-3-methyl-glutaryl-coenzyme A reductase [43], while clomazone (CLZ) and fosmidomycin (FSM) selectively block 1-deoxy-d-xylulose 5-phosphate synthase (DXS) [54] and 1-deoxy-d-xylulose 5-phosphate reductoisomerase (DXR) [46], respectively, the first and second enzymes of the MEP pathway (Fig. 1). Norflurazon (NFZ) has been widely utilized as a specific inhibitor of the downstream enzyme phytoene desaturase [11] due to its ability to isolate regulation of the MEP pathway from that of carotenoid biosynthesis, a principal sink for MEP pathway derived IDP and DMADP in chloroplasts.

While the MVA pathway yields IDP and DMADP primarily for the production of sesquiterpenoids, phytosterols, brassinosteroids, polyprenols, and ubiquinone [84], most if not all terpenoid-derived co-factors required for photosynthesis utilize IDP and DMADP provided by the MEP pathway. For instance, the MEP pathway contributes building blocks for the biosynthesis of photosynthetic pigments including carotenoids and chlorophyll [70] and the electron transport co-factors phylloquinone [89] and plastoquinone [49] (Fig. 1). However, the link between IDP and DMADP availability, flux through the MEP pathway, and photosynthetic carbon assimilation has not been well established. Part of this deficiency in understanding relates to the limited information regarding the proportion of total fixed carbon dedicated to isoprenoid metabolism for the synthesis of photosynthetic pigments in the chloroplast. Metabolic flux through the MEP pathway has recently been reported in model [90] and non-model plant systems [32, 61]. Yet such studies have so far not calculated the measured flux through this pathway as a function of the total assimilated carbon budget. Such information would be highly valuable for clarifying the role of isoprenoid precursor availability in supporting photosynthesis as well as the impact of photosynthesis on flux through the MEP pathway.

Here we describe a method for estimating carbon assimilation in plants that does not require real time gas exchange measurements, is flexible enough to be used on small plants, and could be implemented on large numbers of plants simultaneously. It is based on elemental analysis—isotope ratio mass spectrometry (EA-IRMS) analysis of ^13^C content in isotopically labeled plant tissue. IRMS is a technique involving a magnetic sector mass spectrometer that measures the ^13^C/^12^C ratio and total C content in a gas or tissue sample with high precision. It may either be fed by an EA combustion oven, which combusts plant tissue to CO_2_ [57] or by the gas flow from a plant cuvette exhaust, which supplies CO_2_ directly to an inline mass spectrometer [15]. Its high sensitivity has made it instrumental in identifying adulterants in agricultural products such as honey [16] as well as detecting performance enhancing drugs in sports [7]. When used in conjunction with gas chromatography (GC), it can provide compound specific isotope analysis [55]. It has been used in the plant sciences to investigate the biosynthetic origins of metabolites such as leaf waxes [67]. Herein we describe a new application of IRMS which takes advantage of the rise in popularity of whole plant ^13^CO_2_ labeling. We demonstrate its similarity to the results obtained from gas exchange measurements and provide proof of principle using *Arabidopsis* mutants and herbicide treatments which target the MEP pathway. Given the role of the MEP pathway in supplying the biosynthesis of photosynthetic pigments and redox co-factors (Fig. 1), we initially postulated that long term deficiencies in the availability of IDP and DMADP might result in reduced photosynthetic efficiency. Using this method, we have instead determined that the dependence of photosynthetic carbon assimilation on flux through the MEP pathway is much more immediate, highlighting the rapid turnover of terpenoid-derived photosynthetic co-factors.

## Methods and materials

### Plant lines and cultivation

*Arabidopsis thaliana* lines designated ‘wild type’ were ecotype Columbia 0 seeds. Mutant lines *hds3*, *dxs3*, and *prl1* have been described previously [28, 63]. The *xpt2* mutant is a T-DNA insertion line (SAIL_378_C01) of AT5G17630. *Arabidopsis* seeds were sown in a mixture of 1:3 perlite:BX soil mixture (Promix) and stratified at 4 °C for 72 h before transfer to an environmentally controlled growth chamber equipped with fluorescent lighting maintained either at standard conditions (21 °C, ~ 60% relative humidity, 140 photosynthetically active radiation [PAR; μEinsteins m^−2^ s^−1^]) or under different light intensities (80, 140, 180, or 500 PAR). Light intensity was verified with a Li-Cor 250A visible light sensor. Mutant lines and plants scheduled for herbicide treatment were all grown under standard conditions. All plants were grown according to a 24 h photoperiod which included 9 h light (short day conditions). All experimental plants were labeled at the rosette stage in the vegetative growth phase before initiation of flowering and watered the day before labeling experiments.

### Inhibitor treatments

Various inhibitor treatments were applied to plants to determine the impact of pharmacological blocks of selected metabolic pathways. Plants were coated with a thin mist of inhibitor spray (~ 5 mL/plant) applied 24 h prior to ^13^CO_2_ labeling. Concentrations and solvents were as follows: 10 μM MEV, 25 μM FSM, 25 μM CLZ, 1 mM chloramphenicol (CAM), 100 μM NFZ. Working solutions were prepared on the same day of application by diluting a 100X stock in 50% (v/v) methanol with water. Control plants were sprayed with the equivalent working solvent (0.5% (v/v) methanol) without herbicides. For DMADP labeling assays, plants were treated with CLZ (25 or 50 μM), FSM (10 or 100 μM), or NFZ (10 or 50 μM) in 0.5% methanol in water, or 0.5% methanol in water only (control) and returned to the growth chamber for 60 min. They were then acclimatized in the flow cuvette for 30 min and labeled for 15 min in an atmosphere containing 400 μL L^−1 13^CO_2_ prior to flash freezing, as described below under “Whole plant labeling experiments”. DMADP labeling in lyophilized, ground leaf tissue was analyzed by gas chromatography–mass spectrometry following phosphoric acid conversion to isoprene as described previously [12]. Methane positive chemical ionization and exact label incorporation calculations were performed as described in [90]. Carbon assimilation trends for herbicide treatments and mutant lines were calculated by linear regression of the data points. Statistical significance was determined by Student’s *t*-tests. P values and confidence intervals were calculated in Microsoft Excel (version 2016) using the Analysis ToolPak.

### Whole plant labeling experiments

**Fig. 2:**
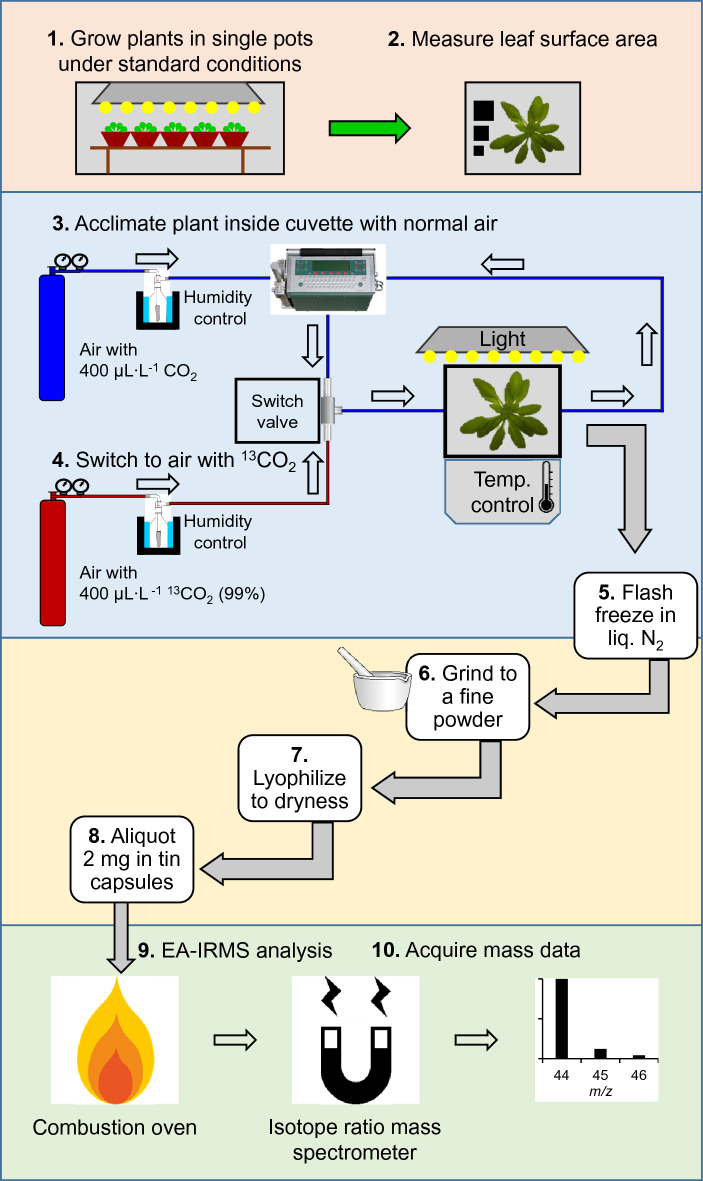
Workflow for whole plant isotopic labeling and quantification of ^13^C label by EA-IRMS. Solid arrows signify the order of steps. Hollow arrows represent gas flow. Both air sources (normal and labeled air) were supplied by compressed air tanks containing 400 μL L^−1^ CO_2_ or ^13^CO_2_ (99% enrichment) in a mixture of nitrogen:oxygen (80:20). Air was (de)humidified by passing through a chilled wash bottle containing water. CO_2_ and H_2_O vapor were quantified before entering the cuvette (reference) and in the cuvette exhaust (sample). After processing labeled plant tissue, 2 mg aliquots were analyzed by EA-IRMS, which consisted of combustion of the sample to carbon dioxide, separation in a magnetic sector mass analyzer, and data acquisition

**Fig. 3:**
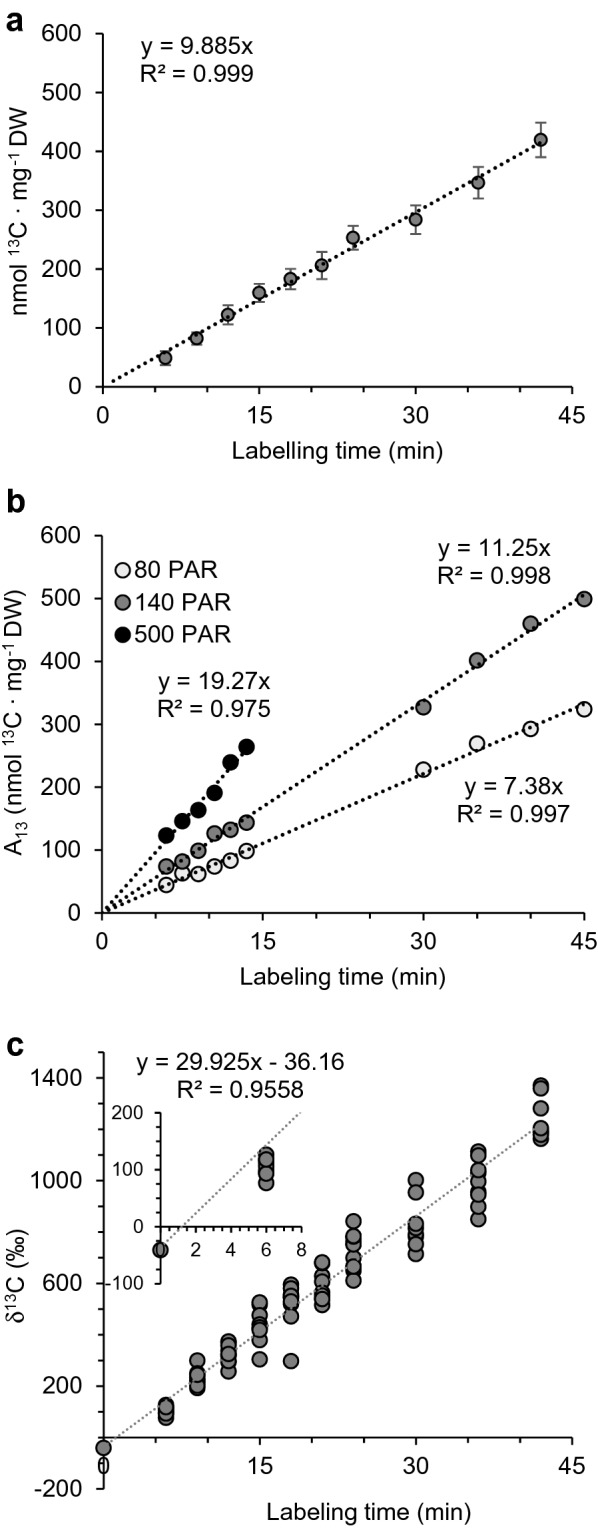
Assimilation of ^13^C during time course labeling assays of wild-type *Arabidopsis* as determined by elemental analysis-isotope ratio mass spectrometry (EA-IRMS). **a** plants were equilibrated in a dynamic flow cuvette under standard conditions (see “Methods and materials” for details) in a natural atmosphere until a photosynthetic steady state was attained, then labeled with 400 μL L^−1 13^CO_2_ before flash freezing in liquid nitrogen. Aliquots of ground, lyophilized tissue were analyzed by EA-IRMS. Naturally occurring isotope abundance was subtracted using the y-intercept of the raw data, which was identical to the natural abundance detected in unlabeled controls. Data points indicate net ^13^C isotope assimilated (A_13_) by individual plants during the labeling experiment. Between 14 and 19 plants were used for each time point as follow: 6 min, n = 15; 9 min, n = 16; 12 min, n = 15; 15 min, n = 15; 18 min, n = 16; 21 min, n = 16; 24 min, n = 19; 30 min, n = 16; 36 min, n = 16; 42 min, n = 14. Error bars show standard deviation. **b** Light intensity was varied to assess the ability of this method to quantitatively describe carbon assimilation under different environmental conditions. Each point represents an individual plant. Time course labeling series were performed on plants cultivated and labeled under low (80 PAR, n = 10), medium (140 PAR, n = 10), or high light conditions (500 PAR, n = 6). Standard conditions were maintained for all other variables. **c** Alternative representation of data in **a** as ^13^C/^12^C isotope ratios where δ^13^C = (R_sample_/R_PDB_ − 1) × 1000, R_sample_ is the ^13^C/^12^C ratio of the sample, and RPDB is the same ratio of the PeeDee Belemnite reference material (0.0112372). The y-intercept value (− 36.16 ‰, see inset) can be used to infer the ^13^C discrimination in *Arabidopsis* and closely matches values obtained for unlabeled control plants, which is within the normal range for a C3 plant

Whole plant, short term ^13^CO_2_ labeling assays were based on previously reported protocols [34, 50] and as described in the schematic workflow (Fig. 2). To establish the linearity of the technique, 14–19 plants were analyzed per time point from 6 to 42 min (Fig. 3a) for a total of 158 individual whole plant labeling experiments. For mutant, herbicide treatment, and light intensity time courses, between 5 and 11 plants were used in each group, as indicated in the corresponding figure legends. Environmental variables (temperature, light intensity, humidity) for labeling in the dynamic flow cuvette were adjusted to be identical to a given plant’s cultivation conditions in growth chambers, based on readings obtained with the same Li-Cor 250A light sensor used to measure light intensity in growth chambers and a TC01 USB thermocouple (National Instruments), which was installed in the flow cuvette and placed in continuous contact with the abaxial leaf surface (Additional file 1: Figure S1). Prior to initiating labeling, all plants were acclimated in a normal atmosphere for a minimum of 30 min at a flow rate of 1.0 L min^−1^ until photosynthesis had stabilized, as judged by gas exchange measurements performed with either a Licor 840a CO_2_/H_2_O analyzer or Licor 6400 photosynthesis measurement system as described previously [90]. Cuvette temperature was maintained at 21 °C and confirmed with thermocouple readings. CO_2_ concentration was maintained at 400 μL L^−1^ based on IRGA sensor readings during acclimation. Once gas exchange measurements in the normal atmosphere had stabilized (< 1% variation over 5 min), labeling was initiated by a single step change to an atmosphere identical to the previous one but with 400 μL L^−1 13^CO_2_ (99% enrichment; Linde Gas). Atmospheric switching was controlled manually with a 3-way switch valve just upstream of the plant cuvette. Harvesting and metabolic quenching were accomplished through freezing in liquid nitrogen, and the uncorrected labeling time was recorded as the time from admitting the labeling atmosphere to the cuvette until plant freezing. Upon switching to the labeling atmosphere containing 400 μL L^−1 13^CO_2_, the atmospheric half-life was determined from the decay of the ^12^CO_2_ signal by calculating the time to reach the midpoint between the ^12^CO_2_ signal prior to initiating labeling and the minimum signal observed in the ^13^CO_2_ containing atmosphere (the IRGA, while tuned to maximize sensitivity to ^12^CO_2_, nonetheless detects ^13^CO_2_ with reduced sensitivity, providing a means of estimating atmospheric half-life under these flow conditions). This value, representing the time to reach a 50:50 mixture of the two atmospheres, was subtracted from the uncorrected labeling times. The corrected labeling times were therefore calculated from the halfway point between atmospheric changeovers until harvest. All plants were thoroughly ground to a fine powder while frozen in liquid nitrogen and lyophilized to dryness prior to analysis.

To measure background isotope abundances, nine wild-type negative control plants were individually acclimated in the chamber and harvested without exposure to the isotopically enriched atmosphere. The background % atom ^13^C values of these unlabeled control plants, as judged by IRMS, were compared to the y-intercept of time course labeling regression lines obtained from raw data and found to be essentially identical. This naturally occurring ^13^C background was subtracted from the raw IRMS data to calculate ^13^C assimilated during the labeling experiment.

### EA-IRMS analysis

Aliquots of approximately 2 mg lyophilized plant tissue were weighed into tin capsules (Elementar Microanalysis) using a Mettler Toledo XP2U microbalance accurate to 10^–7^ g and their exact weights recorded. EA-IRMS was performed by the Utah State University Stable Isotope Laboratory (Logan, UT). The samples were analyzed for total C and ^13^C abundance using continuous flow direct-combustion and IRMS with a PDZ Europa Scientific ANCA 20–20 system (Sercon Ltd., Cheshire, U.K.). The flow rate of high purity He carrier gas was 90 mL min^−1^ and an 18 s pulse of ultra-high purity O_2_ was used for sample combustion. Temperature settings were as follows: combustion furnace, 969 ºC; reduction furnace, 595 ºC; GC oven, 65 ºC. Electronic settings included the following: electron current 200 μA, ion repeller 4.8 V, ionization energy 99 eV, ion focus 83 V, and high tension (HT) at 2469 V. During tuning, HT was adjusted to the center of the 2/1 beam ratio plateau following injection of pure CO_2_ gas (800 μg C) at natural abundance ^13^C. With these settings, integration of sample N_2_ peaks (masses 28, 29, 30) occurred between 91 and 190 s and integration of sample CO_2_ (representing all C) peaks (masses 44, 45, 46) occurred between 230 and 440 s. Samples were run versus a glucose standard containing 950 μg C at 1.10068 atom % ^13^C. Precision, as judged by deviation from expected ^13^C values observed in the glucose standard, was better than 0.1‰ ^13^C. To test the reproducibility and precision of the complete analytical pipeline, an *Arabidopsis thaliana* wild-type control plant labeled under a ^13^CO_2_ atmosphere for 1 h was weighed out as described above and analyzed ten times.

## Results

### Isotope based estimates of carbon assimilation closely parallel gas exchange values

Rosette stage *Arabidopsis* plants were subjected to physiological ^13^CO_2_ labeling in a dynamic flow cuvette following adaptation in a normal atmosphere, during which time gas exchange measurements were taken. At the end of a pre-determined labeling period lasting from 6 to 42 min, the plants were flash frozen in liquid nitrogen. When aliquots of the powdered, lyophilized tissue were subjected to EA-IRMS analysis, a linear accumulation of ^13^C was observed over time (Fig. 3a), indicating that the rate of ^13^C assimilation could be obtained from the resulting slope (9.89 nmol ^13^CO_2_ mg^−1^ D.W. min^−1^). When plants were grown and labeled at a range of light intensities, the slope increased with light intensity as expected, indicating that ^13^C content was a reliable indicator of differential assimilation rates (Fig. 3b).

For plants exposed only to a normal atmosphere, where the ^13^C abundance is approximately 1.1%, the ^13^C content is typically depleted in plant tissue to an extent which reflects a combination of discrimination processes, including the discrimination of Rubisco against ^13^CO_2_ and a decreased diffusion constant of the heavier isotope in the gas through the stomata and the cytosol as it enters the chloroplast. This depletion is often represented as δ^13^C (expressed in ‰ and calculated as 1000 × (R_sample_ – R_standard_)/R_standard_) and usually falls in the range of − 22 to − 35‰ for a C3 plant such as *Arabidopsis* [57]. We calculated δ^13^C values for time course labeled plants shown in Fig. 3a; however, as expected, after less than 1 min in an atmosphere containing 400 μL L^−1 13^CO_2_, the δ^13^C levels surpassed the natural isotopic abundance and increase to upwards of 1400‰ after nearly 42 min of continuous labeling (Fig. 3c). The calculated y-intercept for the computed linear regression of these data was − 36.16‰, which closely matched δ^13^C values measured in unlabeled control plants.

We estimated intra-replicate variability of this approach by carrying out multiple IRMS analyses of % ^13^C and total C content on a control sample which had been ^13^CO_2_ labeled for ~ 1 h. The % ^13^C in this sample was measured at 4.70009 ± 0.01105% with a relative standard deviation of 0.7% (n = 10). Instrumental precision based on labeled plant standards was measured at 0.07%. Most of the error, while minimal, was likely incurred during the weighing step.

**Fig. 4:**
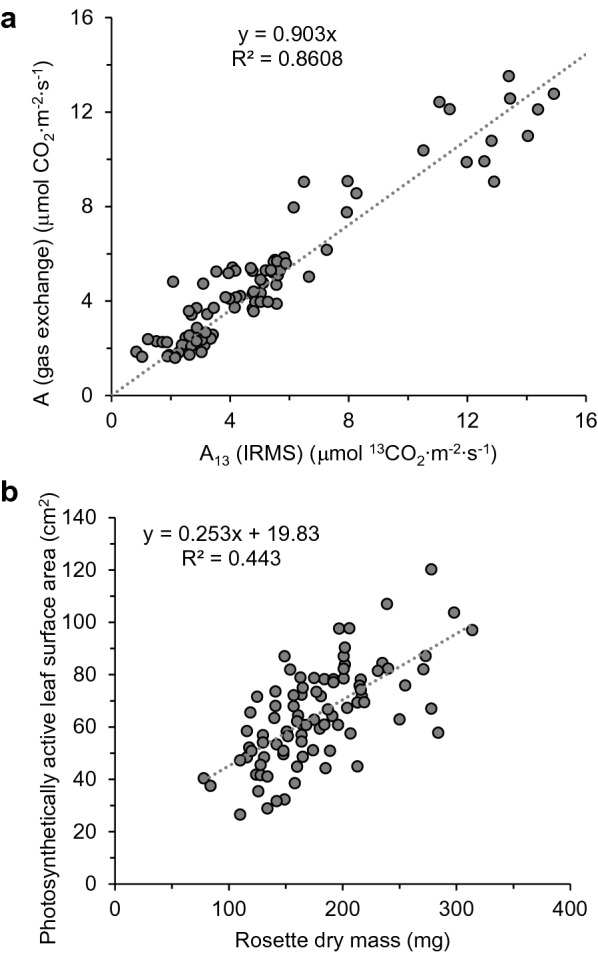
Net carbon assimilation values (A) obtained from isotope ratio mass spectrometry (IRMS) of ^13^C labeled plant tissue are quantitatively similar to those obtained by gas exchange measurements. **a** Two methods to calculate A in time course labeled *Arabidopsis* plants adapted to different light intensities (80–500 PAR, n = 107 plants). Gas exchange measurements of cuvette enclosed plants were taken continuously during the 30–45 min pre-labeling adaptation phase, and reported values represent the average A in normal air for the 3 min prior to introducing the ^13^CO_2_-containing atmosphere. For each plant, the analogous IRMS-based carbon assimilation estimate was calculated from the raw IRMS data (μg ^13^C mg^−1^ D.W.) and converted to μmol ^13^CO_2_ m^−2^ s^−1^ based on their individual leaf surface areas, rosette dry weights, and labeling times. Outliers were identified by the interquartile range rule. **b** The correlation between surface area and dry mass of *Arabidopsis* rosettes is weak and insufficient to establish a general rule used in gas exchange and ^13^C labeling experiments. Surface area was estimated by comparison to a calibrated size standard as described in methods. Mass was determined following lyophilization of the intact rosette. Each point represents a single rosette stage *Arabidopsis* plant 50–70 days old. Outliers were removed according to the interquartile range rule

Although this IRMS-based approach to estimating carbon assimilation calculates assimilated ^13^C on a “per dry weight” basis, we used additional metrics collected for the samples in Fig. 3b (total rosette dry weight and photosynthetically active surface area) to convert these values to those used in gas exchange, i.e. μmol CO_2_ m^−2^ s^−1^ (Fig. 4a). This conversion was done on an individual basis since the correlation between rosette dry mass and photosynthetically active surface area we observed across ~ 90 samples was too weak to apply a general rule for interconverting mass with surface area (R^2^ = 0.443) (Fig. 4b). After calculating the total amount of ^13^C label in a given plant and dividing this by the calculated leaf surface area and labeling time, we converted the initial units obtained by IRMS analysis (μg ^13^C g^−1^ D.W.) to those used in gas exchange (μmol CO_2_ m^−2^s^−1^). In this fashion, we were able to compare carbon assimilation rates between the two methods using identical units for plants labeled over a range of light intensities (80, 140, or 500 PAR) for which we expected a range of carbon assimilation rates. When paired assimilation data derived from gas exchange and IRMS were plotted for each sample, the resulting slope was close to 1 (0.903; 95% confidence interval (CI) [0.8652, 0.9408], n = 89) (Fig. 4a), demonstrating that the IRMS-based method yielded highly similar results in terms of net carbon assimilation when compared to the gas exchange values for the same samples. The slope of the relationship is expected to be slightly less than 1, representing the loss of unlabeled carbon measured during the adaptation phase of the experiment that is immediately released as CO_2_ and not maintained in the biomass.

### Mutants affected in the MEP pathway or supply of its substrates have suppressed photosynthetic rates

**Fig. 5:**
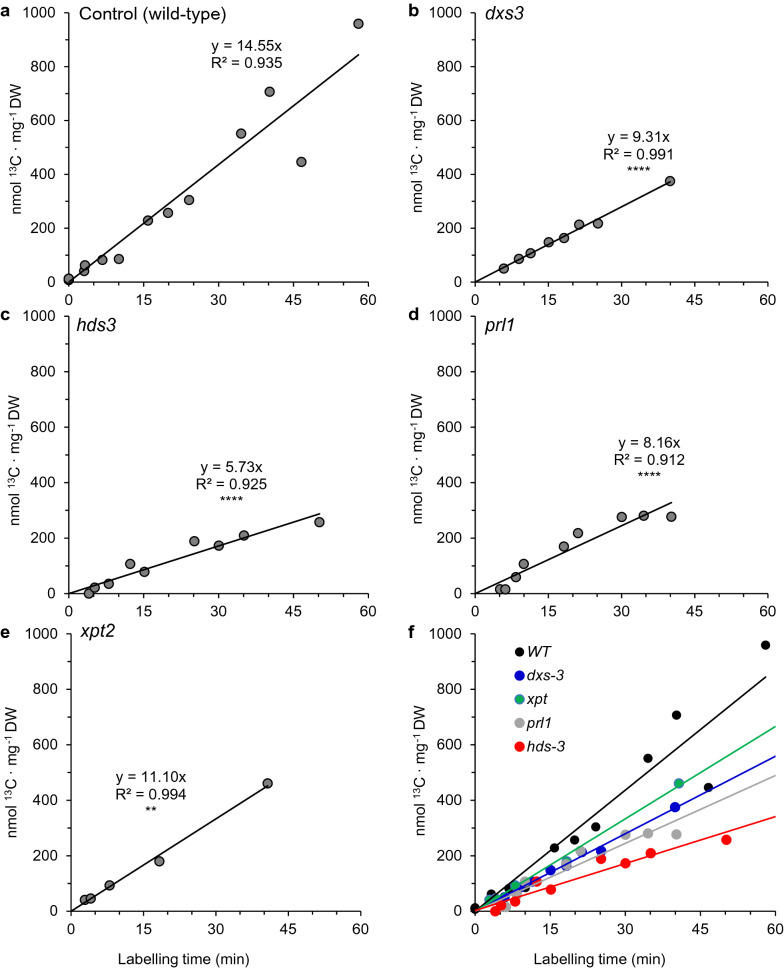
Assimilation of ^13^C by *Arabidopsis* MEP pathway mutants as determined by IRMS of ^13^CO_2_ time-course labeled whole plants (400 μL L^−1^). Each point represents a single intact plant (sample sizes shown in parenthesis) **a** wild-type (n = 10) and **b** *dxs-3* (n = 8), **c** *hds-3* (n = 9), **d** *prl1* (n = 9), and **e** *xpt2* (n = 5) mutant lines, combined in **f**. *P*-values are based on a Student’s *t*-test of the slopes compared to the control: **P < 0.01 and ****P < 0.0001

Having established the suitability of this IRMS-based approach to estimate carbon assimilation, we next applied this technique to a collection of *Arabidopsis* wild-type and mutant lines affected either in structural genes of the MEP pathway or genes related to the transport and supply of substrates for this pathway. Compared to wild-type plants, plants defective in the XYLULOSE-5-PHOSPHATE TRANSPORTER*2* gene (*xpt-2*) displayed significantly diminished carbon assimilation rates (24% lower than wild-type; 95% CI [18%, 29%], n = 5) (Fig. 5). The *prl1* mutant, defective in the *PLEIOTROPIC REGULATORY LOCUS* gene, is indirectly involved in regulating supply of substrate entering the MEP pathway [28]. IRMS analysis showed a decrease in ^13^C assimilation of 44% in this mutant line (95% CI [36%, 52%], n = 9). However, the inhibition of photosynthesis was even more evident in partial loss of function mutants affected in MEP pathway structural genes such as *dxs-3* or *1-hydroxy-2-methyl-2-(E)-butenyl 4-diphosphate synthase 3* (*hds-3*), where the IRMS-based carbon assimilation rate was 36% (95% CI [33%, 39%], n = 8) and 61% (95% CI [55%, 66%], n = 9) reduced, respectively, compared to wild-type plants (n = 10) (Fig. 5) as determined by a Student’s *t*-test of the linear regressions from ^13^C assimilation rates [40]. Taken together, mutants affected in the MEP pathway demonstrated larger declines in assimilation rates than mutants affected in substrate transport and supply. However, we could not rule out that long term, secondary developmental effects of these mutations could also be partly responsible for the observed impact on photosynthesis.

### Herbicides inhibiting chloroplast terpenoid metabolism phenocopy MEP pathway mutants

**Fig. 6:**
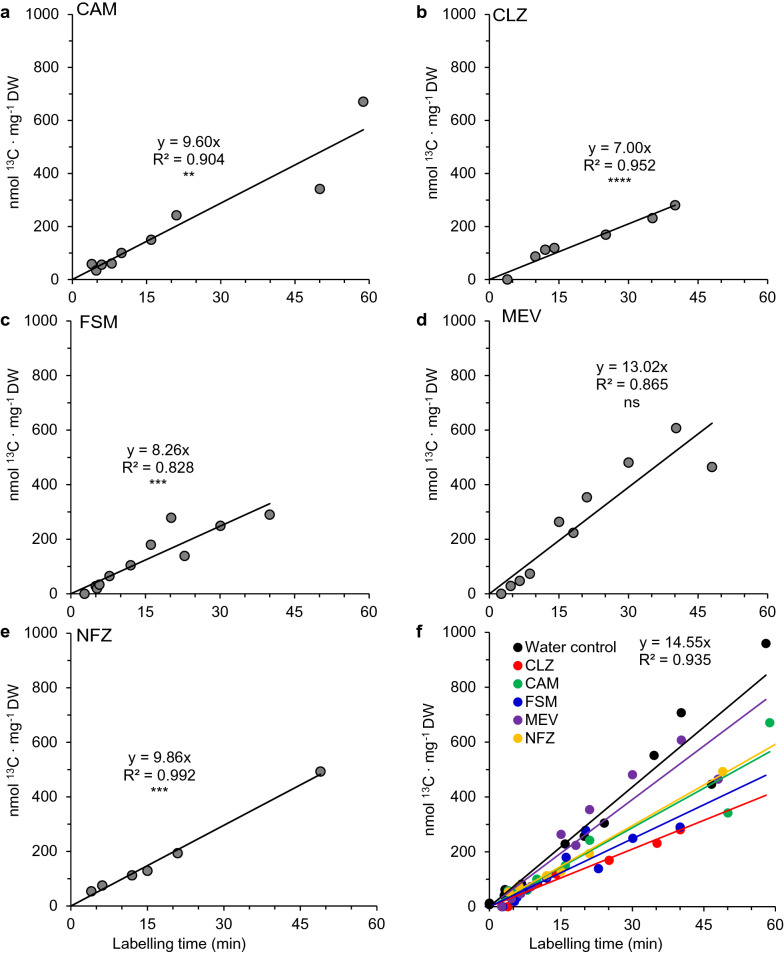
Assimilation of ^13^C by herbicide treated *Arabidopsis* wild-type as determined by IRMS of ^13^CO_2_ time-course labeled whole plants (400 μL L^−1^). Twenty-four hours prior to labeling, plants were treated with either **a** CAM (n = 9), **b** CLZ (n = 7), **c** FSM (n = 11), **d** MEV (n = 10), **e** NFZ (n = 6), combined in **f**. For controls, see Fig. 5a. Each point represents a single, intact plant. *P*-values are based on t-test of regression slopes compared to the control: ns, *P* ≥ 0.05; *P < 0.05; **P < 0.01; ***P < 0.001; ****P < 0.0001

We next explored the role of the MEP pathway in supporting the biosynthesis of photosynthetic co-factors in the short term by treating wild-type plants with CLZ or FSM, which selectively block the first (DXS) and second (DXR) step of the MEP pathway, respectively. Plants were subjected to the same time course labeling experiments 24 h after treatment, and the resulting lyophilized tissue was similarly analyzed by EA-IRMS. As controls, we also treated plants with MEV (which blocks HMG-CoA reductase in the cytosolic mevalonate pathway and exerts a similar effect on the cytosolic pool of IDP and DMADP), NFZ (which blocks the phytoene desaturase step of carotenoid biosynthesis), CAM, which blocks plastidic protein synthesis, or water (control). We hypothesized that CLZ and FSM treatment prior to labeling would simulate the reduced availability of IDP and DMADP in the chloroplast imposed by mutation in structural genes but would not have the same developmental effects resulting from these mutations or long term effects related to reduced levels of photosynthetic pigments or co-factors available to participate in photosynthesis. Compared to wild-type plants treated only with water (Fig. 5a), CAM treated plants displayed a 34%, reduction in carbon assimilation rates (95% CI [22%, 46%], n = 9) (Fig. 6a). Plants treated with CLZ or FSM had reductions of 52% (95% CI [46%, 57%], n = 7) and 43%, (95% CI [32%, 54%], n = 11), respectively (Fig. 6b, c). However, when the cytosolic MVA pathway was blocked with MEV (n = 10), no significant reduction in assimilation rate was observed (Fig. 6d). This suggested the inhibition of photosynthesis observed in mutant plants and wild-type plants treated with MEP pathway inhibitors was specifically related to IDP and DMADP supply in the chloroplast.

**Fig. 7:**
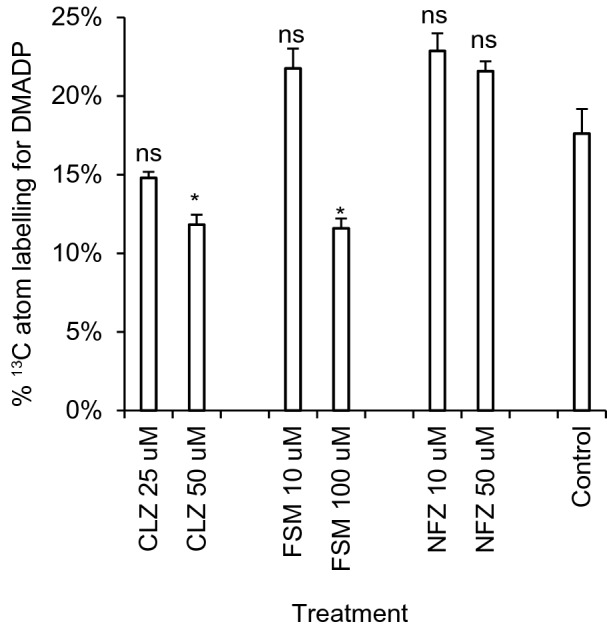
Labeling of DMADP pool in *Arabidopsis* plants treated with MEP-pathway directed herbicides. Clomazone (CLZ) and fosmidomycin (FSM), which target the MEP pathway, cause significant reductions in flux into DMADP in plants incubated in an atmosphere containing 400 μL L^−1 13^CO_2_ 90 min following treatment. In contrast, norflurazon (NFZ), which targets the downstream carotenoid biosynthetic enzyme phytoene desaturase, did not show decreases in ^13^C incorporation compared to controls. N = 3 for each group. *P*-values are based on Student’s two-tailed *t*-test: ns, *P* ≥ 0.05 and *P < 0.05

NFZ treatment also provoked a significant decline in carbon assimilation (32%; 95% CI [28%, 37%], n = 6) (Fig. 6e), although not as severe as herbicides targeting the MEP pathway. Therefore, we next carried out additional short term herbicide treatments (90 min) with CLZ, FSM, and NFZ to confirm that CLZ and FSM (but not NFZ) impacted flux in the MEP pathway under our experimental conditions. Label incorporation into DMADP was measured in the resulting tissue via acid hydrolysis to isoprene gas as described previously [12]. We observed significant reductions in flux towards DMADP in CLZ and FSM treated plants (particularly at the highest concentrations employed) but no significant decrease in NFZ treated plants (Fig. 7).

## Discussion

### IRMS-based measures of carbon assimilation offer complementary features compared to gas exchange

Despite the centrality of gas exchange measurements in elucidating the mechanisms of photosynthetic carbon assimilation, there are limitations to this technique when working with the model plant *Arabidopsis*. For instance, many mutants of *Arabidopsis* affected in some aspect of the carbon reduction cycle may present a stunted growth phenotype which assimilate too little carbon to quantify gas exchange parameters reliably. Applying leaf clamps may not be physically feasible in this case, and *Arabidopsis* leaf surface area is instead estimated graphically. However, the partially overlapping leaves of *Arabidopsis* rosettes, whose extent increases as the plant develops, do not lend themselves well to accurate calculations of leaf surface area. In addition, gas exchange in general is a laborious process poorly suited to screening large numbers of plant lines.

For these reasons, we evaluated IRMS-based estimates of carbon assimilation in a collection of time course ^13^CO_2_ labeled plants to facilitate measurement of gas exchange in *Arabidopsis* and found that this approach provides the following advantages. First, it is based on tissue mass rather than leaf surface area, which avoids the uncertainty of partial leaf overlap and differences in individual leaf assimilation rates based on partial or total shading. Partially shaded leaves carry out photosynthesis at a lower rate which is otherwise difficult to account for. Gas exchange as a technique originated in combination with leaf clamps on large leaf species such as maize but performs poorly for small rosette leaves, where leaf surface area must be estimated graphically. These problems were largely overcome by basing carbon assimilation on mass of dry tissue, which provided a highly linear (R^2^ > 0.999) measure of assimilation (Fig. 3a) whose δ^13^C curve coincided with the expected depletion of ^13^C isotopes in unlabeled plants (Fig. 3c). The agreement between the δ^13^C measured in unlabeled *Arabidopsis* tissue and the y-intercept value obtained by linear regression of time course labeled plants supported the notion that IRMS-based estimates of carbon assimilation in ^13^C labeled plants provide physiologically meaningful measures of photosynthetic rates.

Second, the analysis described here is post hoc, e.g. it can be performed after the fact with previously labeled samples if stored properly and requires no online detection system. Even ^13^C content in older or partially degraded samples can be measured accurately since EA-IRMS uses combustion to convert organic matter to CO_2_ prior to entering the mass analyzer. Third, if the ^13^C IRMS data are to be correlated to targeted analysis of label in metabolite pools, it is convenient that assimilation rates and labeling of target metabolites be based on incubation in the same ^13^CO_2_ atmosphere. Previously reported labeling techniques involve measuring gas exchange in a normal atmosphere and then switching to a new atmosphere for the labeling portion of the experiment [90]. This may inadvertently introduce undesirable changes to the plant’s metabolism. The approach described herein permits carbon assimilation estimates and post-harvest targeted analysis to be correlated without the assumption that the change between an unlabeled and labeling atmosphere do not induce metabolic changes.

Finally, small *Arabidopsis* mutants or young plants which cannot physically undergo leaf clamping or enclose enough area to produce a sufficient CO_2_ drawdown (as measured on an IRGA) can nonetheless be labeled and analyzed by EA-IRMS to estimate carbon assimilation using the technique described here. Plate grown seedlings as young as 10–14 days could theoretically be labeled and analyzed with this technique if grown at a sufficient density on sterile plates to yield enough tissue for EA-IRMS analysis. Moreover, large biological replicate pools can be labeled simultaneously in a sufficiently large cuvette, increasing the throughput of labeling experiments and improving the statistical power of analysis. In this fashion, this technique can be used to screen multiple lines or treatments in parallel based on comparative carbon fixation rates under a given condition. It should be noted that, by design, the data presented here only address leaf level carbon fixation and do not account for carbohydrates transported to root tissue. However, the transport of ^13^C into root tissue may be monitored with exactly the same approach by harvesting root tissue separately and subjecting aliquots to EA-IRMS. Indeed, this technique may provide a powerful method for analyzing the partitioning of carbon resources by comparison of ^13^C detected in leaf and root tissue separately.

The use of IRMS to investigate photosynthesis has several precedents. IRMS analysis of carbon isotopes in leaf tissue has previously been used to determine the influence of CO_2_ partial pressure across stomata on isotopic discrimination during carbon assimilation [25]. In this study, which relied on natural ^13^C abundances rather than labeling, changes in carbon isotope composition of CO_2_ passing over the surface of a leaf were correlated to isotopic discrimination in intact leaves as the CO_2_ partial pressure was experimentally varied. It confirmed that ^13^C discrimination increased as the CO_2_ partial pressure gradient across stomata decreased in C_3_ plants but not in C_4_ plants. IRMS analysis has also been used to probe the source of carbon supplying isoprene emissions in myrtle, buckthorn, and velvet bean [1], where δ^13^C differences in CO_2_ and isoprene emissions implicated DXS as the main isotopic discrimination step in isoprene formation. IRMS analysis was further used to identify the presence of carbon pools contributing to dark respiration not derived from recent photosynthate [59]. To our knowledge, our method is the first report using ^13^CO_2_ and IRMS to estimate carbon assimilation directly.

### IRMS-based estimates of carbon assimilation closely resemble those of gas exchange measurements

To compare assimilation rates estimated by ^13^C content to traditional gas exchange measurements, we converted ^13^C content on a per mg basis to the units used in gas exchange (μmol CO_2_ m^−2^ s^−1^). The observed slope, slightly less than 1, reflects unlabeled carbon loss released during the labeling segment. This release is comprised of carbon within the photorespiratory pool, additional pools lost from the C2 cycle, and related processes termed non-photorespiratory CO_2_ release [76, 77, 83]. In a normal atmosphere, this CO_2_ release is included in A. However, once the atmosphere is switched to a ^13^CO_2_ containing atmosphere, the slow loss of residual, unlabeled CO_2_ is no longer subtracted from the apparent net assimilation of ^13^C (A_13_) since we include only ^13^C quantified by EA-IRMS in our analysis. Therefore, the same time points yield slightly higher values for A_13_ than for A, and consequently, the slope remains below 1. This slope remained consistent over all time point assayed, suggesting the slow release of unlabeled CO_2_ from non-photorespiratory sources continued throughout the ~ 45 min labeling period. In contrast, photorespiratory intermediates are expected to become fully labeled within minutes, and their loss as ^13^CO_2_ would then be factored into the ^13^C detected in lyophilized plant tissues. Our results support previous observations that non-photorespiratory CO_2_ loss is a significant and distinct form of respiration than photorespiratory CO_2_ loss [59].

### A block in the MEP pathway disrupts photosynthesis through multiple mechanisms

We observed a strict decline in carbon assimilation when the MEP pathway was blocked with CLZ or FSM for 24 h (Fig. 6), and the rapid inhibition of the MEP pathway reported elsewhere for these herbicides was confirmed here by monitoring flux into DMADP shortly after treatment (1.5 h) (Fig. 7). While FSM acts as a competitive inhibitor of DXR, CLZ is converted *in planta* to ketoclomazone, which acts as an uncompetitive DXS inhibitor with respect to pyruvate but a mixed inhibitor with respect to d-glyceraldehyde-3-phosphate [54]. These herbicide inhibitor results were similar to the assimilation rates observed in partial loss of function mutant *hds-3* [33] (Fig. 5c) as well as in the temperature sensitive mutant of *DXS* originally named *chill sensitive 5* (*chs-5*, [3]) (later renamed *dxs-3* [63]) (Fig. 5b), confirming that both a pharmacological and genetic block in the MEP pathway produce similar results. CAM treatment serves as positive control for inhibiting photosynthesis as the gene encoding the large subunit of Rubisco, *rbcL*, is encoded in the plastid genome of land plants and algae [13]. The dependence of photosynthesis on the MEP pathway is well established due to its roles in supplying the precursors for chlorophyll and carotenoid biosynthesis [70], but the exact cause of photoinhibition and lethality under conditions of MEP pathway inhibition are less clear. A reduction in photosystem II chlorophyll fluorescence, Rubisco carboxylase activity, photosynthetic electron transport, and carbon assimilation were previously reported in plants one hour after treatment with FSM [64]. This is consistent with the essential role of DXR in supplying IDP and DMADP for pigment synthesis as demonstrated in transgenic *Arabidopsis* lines up and down regulating this gene [18].

Carotenoids and chlorophylls are continuously synthesized and degraded during the day [19], and a block in their synthesis is expected to noticeably impact carbon assimilation rates within hours to days if the degradation pathway remains active. ^14^CO_2_ pulse-labeling experiments resulted in detection of radiolabel in β-carotene and chlorophyll a in as little as 30 min [8], underscoring the short half lives of these pigments exposed to high doses of radiation and the dependency on MEP pathway flux to maintain carbon assimilation rates. FSM inhibition of photosynthesis could be relieved by insertion of the complete MVA pathway into the tobacco chloroplast genome, providing an alternative source of IDP and DMADP for GGPP synthesis that was not susceptible to FSM inhibition [45]. However, FSM photoinhibition likely results from a combination of depleted pigment reserves as well as an accumulation of phototoxic intermediates of chlorophyll biosynthesis, which relies on equal contributions from the tetrapyrrole and phytyl diphosphate pathways. When the MEP pathway is blocked by FSM, a shortage of available GGPP for phytyl diphosphate production causes an accumulation of free tetrapyrrolic intermediates in the chlorophyll pathway which induces the formation of singlet oxygen and causes photooxidative stress [44]. Kim et al. further demonstrated that this FSM toxicity could be reversed by phytol supplementation or a chemical or genetic block in tetrapyrrole biosynthesis, suggesting the surplus of photoreactive tetrapyrroles in phytol-deficient cells is the source of toxicity in the short term. Indeed, *Arabidopsis* mutants which accumulate protochlorophyllide display a FSM-poisoning phenotype related to photooxidative stress when transitioned to the light [56], underscoring the requirement for careful coordination of these two pathways during chlorophyll biosynthesis. NFZ, in contrast, is expected to impact carotenoid levels without directly disrupting tetrapyrrole/phytol ratios because this herbicide targets phytoene desaturase downstream of the MEP pathway and should not, in principle, affect chloroplast pools of IDP and DMADP. This should therefore result in a lesser degree of photoinhibition compared to a block in the MEP pathway as seen in CLZ and FSM treated plants. Consistent with this prediction, these NFZ treatment results suggested that a disruption in supply of carotenoid precursors downstream of IDP and DMADP, while essential to a functional photosynthetic apparatus, was not as detrimental as blocking the MEP pathway, which supplies precursors for multiple photosynthetic components, including chlorophylls, and did not impair flux through the MEP pathway (Fig. 7). We therefore considered that a disruption in MEP pathway flux provoked inhibition of photosynthesis through multiple mechanisms. The phototoxic potential of excess tetrapyrrolic intermediates may explain their role in negative feedback downregulation of the tetrapyrrole biosynthetic pathway [73] and implication in chloroplast-to-nucleus retrograde signaling to coordinate the metabolic status of chloroplasts with the synthesis of nuclear encoded chloroplast-localized proteins [20]. In summary, our results in FSM, CLZ, and NFZ treated plants likely reflect the combined effects of short term (~ 1 h) imbalances in tetrapyrrole/phytol ratios for chlorophyll biosynthesis and/or longer term (> 24 h) impacts on carotenoid and chlorophyll steady state levels.

### Exchange of common isoprenoid intermediates is insufficient to rescue photoinhibition caused by a block in the MEP pathway

Our results may be interpreted in light of the well studied exchange of common intermediates between the MEP and MVA pathways. Since the elucidation of the MEP pathway [68], attempts to establish exchange of IDP and DMADP between the cytosolic and plastidic compartments have relied on a combination of inhibitors and isotopically labeled advanced precursors for each pathway [31, 41, 47, 62]. While many examples of terpenoid secondary metabolites containing isoprenoid units of mixed origin have been documented [23, 37, 38, 66, 85], this exchange appears limited to specialized structures and metabolic contexts and is generally unable to reverse the lethality of a complete genetic or pharmacological block [69]. However, some cytosol-to-plastid exchange of common intermediates for phytohormone or carotenoid synthesis may occur in etiolated seedlings during the transition from skotomorphogenic to photomorphogenic development [43, 60]. While in vitro evidence of a proton symporter capable of facilitating the plastid-to-cytosol unidirectional transport of IDP and GDP was described in spinach, kale, and mustard [9], to date no gene for such a transporter has been isolated from any plant species. Our IRMS-based carbon assimilation results on plants treated with FSM or CLZ are consistent with the notion that any contribution from the MVA pathway to carotenoid or chlorophyll biosynthesis in adult plants is, at most, minor and insufficient to meet the demand for precursors needed to form light harvesting pigments in the chloroplast. Moreover, due to the marked declines in carbon assimilation in FSM or CLZ treated plants we report here and the decrease in fixed carbon resources this implies, it is unsurprising that a block in the MEP pathway should result in a decline in cytosolic terpenoid biosynthesis, though this is not necessarily indicative of exchange of common intermediates.

A more significant form of exchange may occur upstream of the MEP and MVA precursor pathways through the oxidative steps of the cytosolic pentose phosphate pathway [76, 77]. The xylulose 5-phosphate (Xu5P) transporter (XPT) imports Xu5P and ribulose 5-phosphate from the cytosol into chloroplasts [24], both of which may join the Calvin-Benson cycle. Incomplete labeling of isoprene emissions has been attributed to the activity of XPT [76], and this transporter also reportedly recognizes DXP [29], although the physiological significance of translocating DXP between the plastid and cytosol is unclear. We observed a statistically significant reduction in carbon assimilation in the *xpt2* mutant (Fig. 5e), suggesting that it indeed plays a role in maximizing plant performance but is not essential for photosynthetic metabolism.

PLEIOTROPIC REGULATORY LOCUS1 (PRL1) is a WD-40 RNA binding protein with multiple roles in immunity and stress tolerance. The *prl1* mutant accumulates higher than normal levels of chlorophyll and carotenoids and is highly resistant to FSM and CLZ despite no detectable changes to MEP pathway transcript or protein levels [28]. It has also been implicated in root stem cell niche activity [42] and in regulation of miRNAs [91]. Due to its apparently higher than normal flux through the MEP pathway and elevated photosynthetic pigment levels, we compared its carbon assimilation rates to partial loss of function mutants of the MEP pathway (*dxs3* and *hds3*). However, all three mutants displayed significant declines in carbon assimilation rate (Fig. 5), indicating that any increase in flux through the MEP pathway in the *prl1* mutant comes at the expense of developmental abnormalities in stem cell organization or disruptions to miRNA processing.

The IRMS-based carbon assimilation measuring technique presented here represents a novel alternative to gas exchange measurements that is well suited to the investigation of central metabolism in *Arabidopsis*, particularly mutant lines with reduced photosynthetic capacities and stunted growth phenotypes or in combination with herbicide treatments intended to dissect regulatory mechanisms controlling metabolism. Unambiguous assignment of carbon assimilation rates in greening seedlings and other early developmental stages, or in leaves vs. roots to address carbon partitioning between organs, can be implemented with minimal modification of this method. When combined with targeted analysis of isotopic tracer by more conventional metabolomics approaches, this method presents many avenues for furthering insights into the basic mechanisms of photosynthesis.

## Supplementary Information

**Additional file 1: Figure S1.** Preparation of *Arabidopsis thaliana* for whole plant ^13^CO_2_ labeling experiments. A, Photosynthetically active surface area was calculated by photographing the rosette against a white background and comparison of the leaf surface to that of size standards, as determined by using the ‘Magic wand’ function in Adobe Photoshop CS5 to quantify pixels of each standard and leaf surface. B and C, examples of single plant labeling cuvettes with light and temperature control used in this study. The switch valve which alternates between ‘normal’ air and ^13^CO_2_-containing air is located just upstream of the cuvette inlet in both cases. Air is sampled by CO_2_ and H_2_O sensors before entering and after exiting the cuvette to calculate gas exchange parameters and confirm a photosynthetic steady state prior to switch to a ^13^CO_2_-containing atmosphere. D, a thermocouple in continuous contact with the abaxial leaf surface monitors leaf temperature during the acclimation and labeling phases of each experiment. Photo credit: M. Phillips.

## Data Availability

All data and material described in this manuscript have been made available. No additional resources beyond data presented here are described in this manuscript.
