# Supplementary material for: Isotope ratio-based quantification of carbon assimilation highlights the role of plastidial isoprenoid precursor availability in photosynthesis
Source: Plant Methods. 2021 Mar 30;17:32. doi: 10.1186/s13007-021-00731-8 (PMC8008545; doi:10.1186/s13007-021-00731-8)
Supplement: Supplementary file 1 — Additional file 1: Figure S1. Preparation of Arabidopsis thaliana for whole plant 13CO2 labeling experiments. A, Photosynthetically active surface area was calculated by photographing the rosette against a white background and comparison of the leaf surface to that of size standards, as determined by using the ‘Magic wand’ function in Adobe Photoshop CS5 to quantify pixels of each standard and leaf surface. B and C, examples of single plant labeling cuvettes with light and temperature control used in this study. The switch valve which alternates between ‘normal’ air and 13CO2-containing air is located just upstream of the cuvette inlet in both cases. Air is sampled by CO2 and H2O sensors before entering and after exiting the cuvette to calculate gas exchange parameters and confirm a photosynthetic steady state prior to switch to a 13CO2-containing atmosphere. D, a thermocouple in continuous contact with the abaxial leaf surface monitors leaf temperature during the acclimation and labeling phases of each experiment. Photo credit: M. Phillips. [file 13007_2021_731_MOESM1_ESM.pdf]

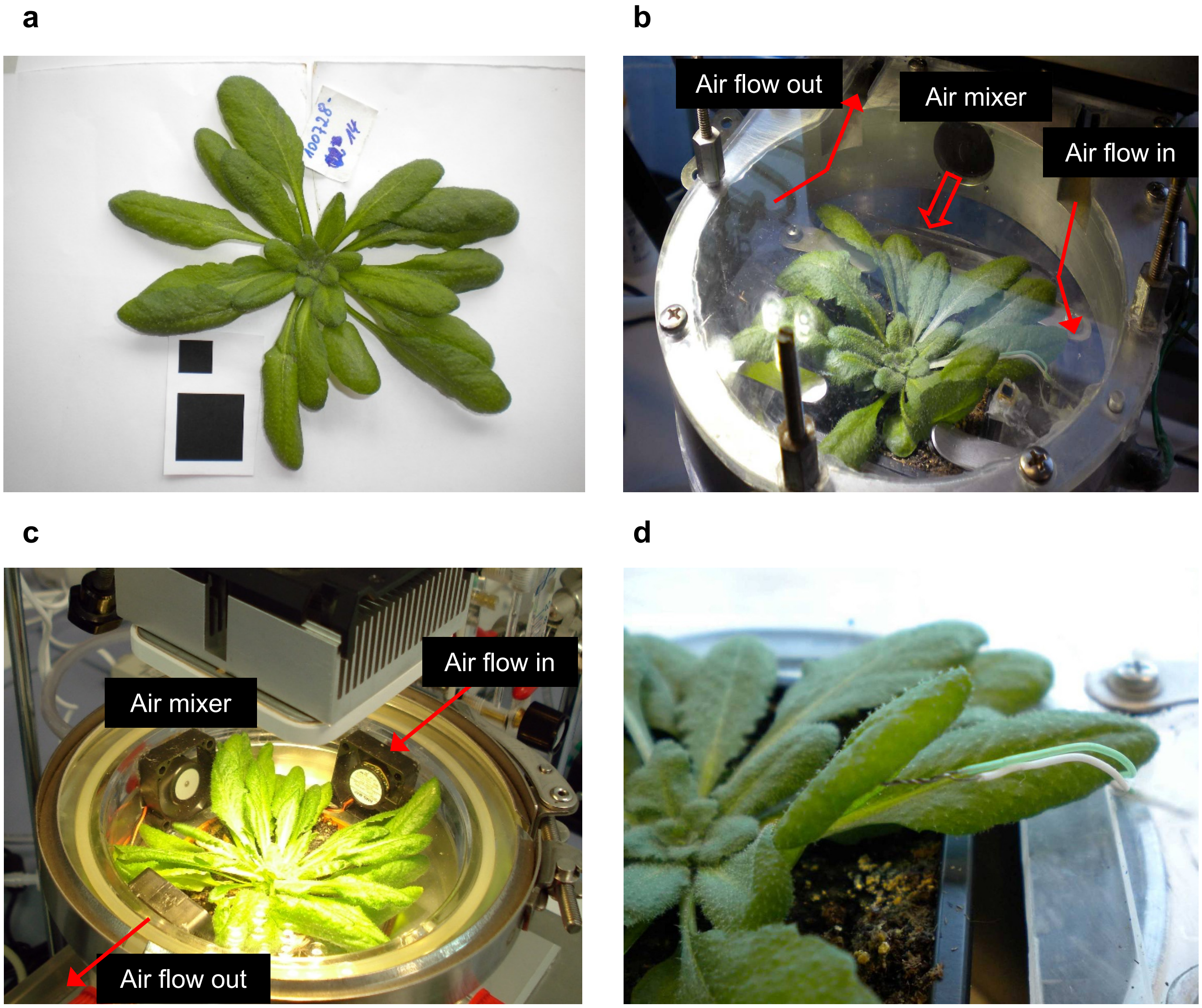

Additional file: figure S1. Preparation of *Arabidopsis thaliana* for whole plant $^{13}CO_2$ labeling experiments. **a**, Photosynthetically active surface area was calculated by photographing the rosette against a white background and comparison of the leaf surface to that of size standards, as determined by using the 'Magic wand' function in Adobe Photoshop CS5 to quantify pixels of each standard and leaf surface. **b** & **c**, Examples of single plant labeling cuvettes with light and temperature control used in this study. The switch valve which alternates between 'normal' air and $^{13}CO_2$-containing air is located just upstream of the cuvette inlet in both cases. Air is sampled by CO_2 and H_2O sensors before entering and after exiting the cuvette to calculate gas exchange parameters and confirm a photosynthetic steady state prior to switch to a $^{13}CO_2$-containing atmosphere. **d**, A thermocouple in continuous contact with the abaxial leaf surface monitors leaf temperature during the acclimation and labeling phases of each experiment. Photo credit: M. Phillips
